# Supplementary material for: The methylome of the model arbuscular mycorrhizal fungus, Rhizophagus irregularis, shares characteristics with early diverging fungi and Dikarya
Source: Commun Biol. 2021 Jul 22;4:901. doi: 10.1038/s42003-021-02414-5 (PMC8298701; doi:10.1038/s42003-021-02414-5)
Supplement: Supplementary file 1 — Supplementary Information [file 42003_2021_2414_MOESM1_ESM.pdf]

Supplementary information for

The methylome of the model arbuscular mycorrhizal fungus, *Rhizophagus irregularis*, shares characteristics with early diverging fungi and Dikarya

Anurag Chaturvedi[1,#,¶], Joaquim Cruz Corella[1,#], Chanz Robbins[1,#], Anita Loha[2], Laure Menin[3], Natalia Gasilova[3], Frédéric G. Masclaux[1], Soon-Jae Lee[1], Ian R. Sanders[1,*]

[1] Department of Ecology and Evolution, University of Lausanne, Biophore Building, 1015, Lausanne, Switzerland

[2] Department of Plant Molecular Biology, University of Lausanne, 1015 Lausanne, Switzerland

[3] Institute of Chemical Sciences and Engineering, Swiss Federal Institute of Technology Lausanne (EPFL), SSMI, Batochime, CH-1015 Lausanne, Switzerland

¶Present address: Environmental Genomics Group, School of Biosciences, the University of Birmingham, Birmingham, B15 2TT, UK

[*]Corresponding author: ian.sanders@unil.ch

[#]AC, JCC, CR contributed equally to this work

Supplementary Notes

Supplementary Note 1: Genome assembly and annotation

Genomes of the six isolates were assembled into contigs and scaffolded, based on an estimated genome size of 150 Mb, with N50 scaffold lengths ranging from 543 kb in A5 to 2.4 Mb in C2 (Supplementary Table S1)[1,2]. Total scaffold counts ranged from 121 in B12 to 392 in A5, with L50 values ranging from 20 scaffolds in C2 to 61 in A5 (Supplementary Table S1). The number of assembled scaffolds did not decrease as estimated sequence coverage increased. For example, 151 scaffolds and a 20X estimated coverage in isolate A1 and 232 scaffolds with a 79X estimated coverage in isolate C3 (Supplementary Table S1). This might also be due to the difficulty in assembling genomes of dikaryotes. BUSCO analysis revealed that genome completeness ranged from 86.9% in A5 to 95.9% in C2, based on detection of complete, single-copy orthologs known to be conserved in fungi (fungi_db09; 290 orthologs) [3].

Single-copy top hit matches of annotated genes from the genome of each isolate ranged from 19071 protein hits in A5 to 20422 in A1. Phylogenetic relationships among the 6 isolates were inferred after aligning 1838 top protein hits commonly shared among all isolates, and the phylogeny confirmed previously observed results using ddRADseq data [4,5]. (Supplementary Figure S1a). Specifically, A5, B12 and A1 clustered separately from C2 and C5, with roughly 0.0019 substitutions per site. (Supplementary Figure S1a). Moreover, the proximity of C2 and C5 confirmed the very high degree of relatedness. Isolate C3 was the most distant with > 0.005 substitutions per site. Although this conservative approach utilized slightly less than 10% of the identified single-copy proteins in each genome, the median sequence length (log10) did not vary significantly among the isolates, ranging from 2.521 in C3 to 2.535 in C2 and C5 (Supplementary Figure S1b; Supplementary Table S2). Using a different approach, other researchers identified 8255 single-copy protein orthologs suggesting that high intraspecific variability exists among *R. irregularis* isolates [6]. Our data agree with the presence of high intraspecific variability although the estimate may, at present, be slightly inflated due to the lack of functionally validated AMF genes. This, combined with an over representation of coding sequences from *in silico* generated gene models deposited in public databases, may skew the perception of lineage-specific gene repertoires, although they exist.

Supplementary Note 2: Structural variation among *R. irregularis* isolates

The number of SVs (> 30 bp) in the genomes of the six *R. irregularis* isolates, as compared to isolate DAOM 197198, was highly variable; being much higher in isolate C3 than in A1 and A5 (Supplementary Figure S3a; Supplementary Table S3). A dendrogram, based on hierarchical clustering of the SV, was concordant with the phylogenetic distance among the isolates based on the phylogeny based on sets of conserved proteins (Figure 1a; Supplementary Figure S3b), although differences in sequencing depth may lead to an underestimation of SV in isolates A1, A5 and B12 (Supplementary Figure S2). Insertions and deletions were much more abundant than inversions and duplications in all six isolates (Supplementary Figure S3a). Median SV length was different among isolates for each type of variation, and large SVs (> 2kb) were detected in all six isolates (Supplementary Table S4).

Repeated sequences are known to directly and indirectly trigger the formation of structural variation through a range of different mechanisms such as non-allelic homologous recombination, fork stalling and template switching or mobile element insertions. Approximately half of the SVs were found to be related to the presence of repeated sequences (Supplementary Figure S3c). In addition, the high frequency of active transposable elements (TEs) in *R. irregularis* has been suggested as a major source of genomic rearrangements and to contribute largely to its intraspecific genetic variability [6]. In order to address this, the occurrence of the breakpoints of SVs in known families of TEs was investigated. Similar results were observed across the six isolates, where DNA transposons and simple repeats were more commonly associated with the presence of SVs (Supplementary Figure S3d, Supplementary Table S5). These results provide a first insight into the contribution of TEs to the existence of large genomic variations among *R. irregularis* isolates.

Unsurprisingly, most of the SV was found to be located in non-coding regions (from 92% in A1, to 96% in C2). However, in this study, the number of genes directly affected by the presence of SVs compared to the model *R. irregularis* isolate DAOM 197198 ranged from 1517 genes in isolate A1 to 13081 genes in isolate C3 (Supplementary Figure S3e; Supplementary Table S6). Gene ontology (GO) enrichment analysis showed that some biological processes and molecular functions were overrepresented in genes harbouring SV in isolates B12, C2, C5, and C3 (Supplementary Data 1). More specifically, processes such as signalling, protein phosphorylation, regulation of P metabolic processes and activation of the MAPK cascade were significantly enriched in isolates C2, C5 and C3 (Supplementary

Figure 3f). Given that these are all involved in P transport and pathways associated with P regulation, this suggests that structural variation can affect regulation of symbiotic effects.

Supplementary Note 3: Structural variation between two *R. irregularis* clones (isolates C2 and C5)

No clear differences could be observed in SV between isolates C2 and C5. Supplementary Figure S3 shows the negligible number of SVs detected between C2 and C5, when using DAOM 197198 as the reference assembly. Mapping reads of C2 and C5 to the *de novo* C2 genome assembly further reduced the number of SVs between C2 and C5. While it is inevitable that some SV will be detected because of sequencing issues or assembly issues, after manually curating putative isolate-specific variants against alignment files in IGV, it was clear that almost all these variants were differentially called as a result of low read support values and/or insufficient coverage. These were, therefore, disregarded (an example is shown in Supplementary Figure S4). Coupled with the fact that Wyss et al. (2016) could not find convincing SNP differences between these two isolates, these results support the hypothesis that these two *R. irregularis* isolates are almost certainly clones as they are genetically indistinguishable.

Supplementary Note 4: Structural variation between nuclei

Structural variation existing within the dikaryon isolate C3 most likely represents structural differences between its two nucleus genotypes. The amount of structural variation (7732 SVs) fell within the range of variation observed between some of the genetically different isolates and isolate DAOM197198 Supplementary Figure 5a). Assuming that this represents SV differences between the two nucleus genotypes, this suggests that some between nucleus differences are as large as SV between some *R. irregularis* isolates. An alternative hypothesis would be that recombination among nuclei, as previously observed in one *R. irregularis* isolate, could lead to within-fungus SV among nuclei [7]. However, no convincing evidence for recombination was observed between nuclei in isolate A4 [7], a clone of C3, making this alternative hypothesis unlikely. Structural variation within C3 exhibited similar properties to those observed among the isolates. The vast majority of SVs were either insertions (3132) or deletions (4436) (Supplementary Figure S5a) and a large proportion were related to the presence of repeated sequences (3426 SVs) (Supplementary Figure S5b). Additionally, DNA transposons were more associated with SVs than other known TE families (Supplementary Figure S5c). Finally, SV between the two nucleus genotypes occurred in a remarkable number of genes (2437) (Supplementary Figure

S5d), although no significant GO terms were enriched. Together, these results suggest that genomic variation occurring among nuclei within the dikaryon isolate C3 is as large, and shares similar characteristics, to that observed between some homokaryote *R. irregularis* isolates.

Supplementary Note 5: 6mA epigenetic signatures differ between nuclei in dikaryons

We analysed whether the two nucleus genotypes in the dikaryon isolate C3 were differentially methylated. We were only able to perform this analysis on gene sequences that we knew were located on different nuclei. Thus, this restricted our analysis to genes that revealed structural differences between the two nucleus genotypes. Of these, 331 genes were methylated. 153 of them lacked 6mA ApT marks in either primary assembly or in the secondary haplotig. The absence of methylation on genes from one nucleus genotype, and the presence in the other, indicates that differential epigenetic gene regulation can occur between nucleus genotypes in dikaron *R. irregularis*. Because we were restricted to only identifying differential methylation occurring in genes between the two nucleus genotypes, at selected sites that differed in their DNA sequence, this represents a very small sub-sample of the total number of methylated genes in the genome. However, at those sites, 46% showed differential methylation suggesting that levels of methylation differences between the two nucleus genotypes are very high.

Supplementary Note 6: The majority of AMF genes involved in P metabolism, P sensing and signalling and homeostasis are under 6mA epigenetic regulation

We performed a targeted assessment of SV and 6mA presence/absence in genes involved in P transport, P metabolism, signalling and homeostasis, P responsive signalling, as well as genes in other pathways shown to participate in P homeostasis in S*accharomyces cerevisiae* or in AMF [8-12]. We also included genes from the PKA, MAPK and Tor signalling pathways that are influenced by changes in inorganic P supply to AMF. Furthermore, we also included both high and low affinity sugar transporters as they have been shown to be associated expression of high-affinity P transporters [13].

We found that high-affinity P transporters (PT3, PT4) and sugar transporters (MST2, MST3) harboured SV but were not methylated (Supplementary Table 7). On the contrary, the low-affinity SPX domain containing P transporters (PHO87, PHO88, PHO90, PHO91) and the low-affinity sugar transporter SUC1 were methylated in all three isolates and MST4 was methylated in two isolates. SPX domain

proteins have been suggested to be key in P transport and metabolism in eukaryotes including AM fungi [9,14]. Remarkably, all the genes that have been identified in previous studies to be involved in inositol polyphosphate synthesis/hydrolysis and P responsive signalling were 6mA methylated, thus, appearing to be under epigenetic control. Such genes are thought to be very important in AMF because they have to take up and regulated levels of P that are much greater than that needed by the fungus alone. The vacuolar transporter chaperones VTC1, VTC 2 and VTC4, involved in polyphosphate synthesis, and which also contain the SPX domain, were also methylated with some differences between in isolate C5 and its clone C2, suggesting isolate specific epigenetic differences of this methylation mark in fine tuning polyphosphate accumulation. Finally, the parts of the of the PKA signalling pathways, that are upregulated in AMF in response to low Pi environments, and the MAPK and Tor signalling pathways, that are down-regulated at low Pi appear to be almost completely methylated, suggesting strong epigenetic control on the regulation of these pathways in the fungus in varying P environments.

Given the critical role of AMF in P transport to plants, the strong methylation of genes involved in almost all components of polyphosphate biosynthesis and release, and P-responsive signalling suggests 6mA plays an important role in P homeostasis. The majority of the 6mA harbouring genes were methylated in all three isolates. Despite this, a few genes exhibited differential methylation among the isolates which may yield a big impact on either on AMF physiology or differential plant growth. Compared to methylation, the occurrence of SV was far less frequent, especially in gene body which is consistent with an expected high conservation of the P transport and metabolism related genes in this species.

Supplementary Figures

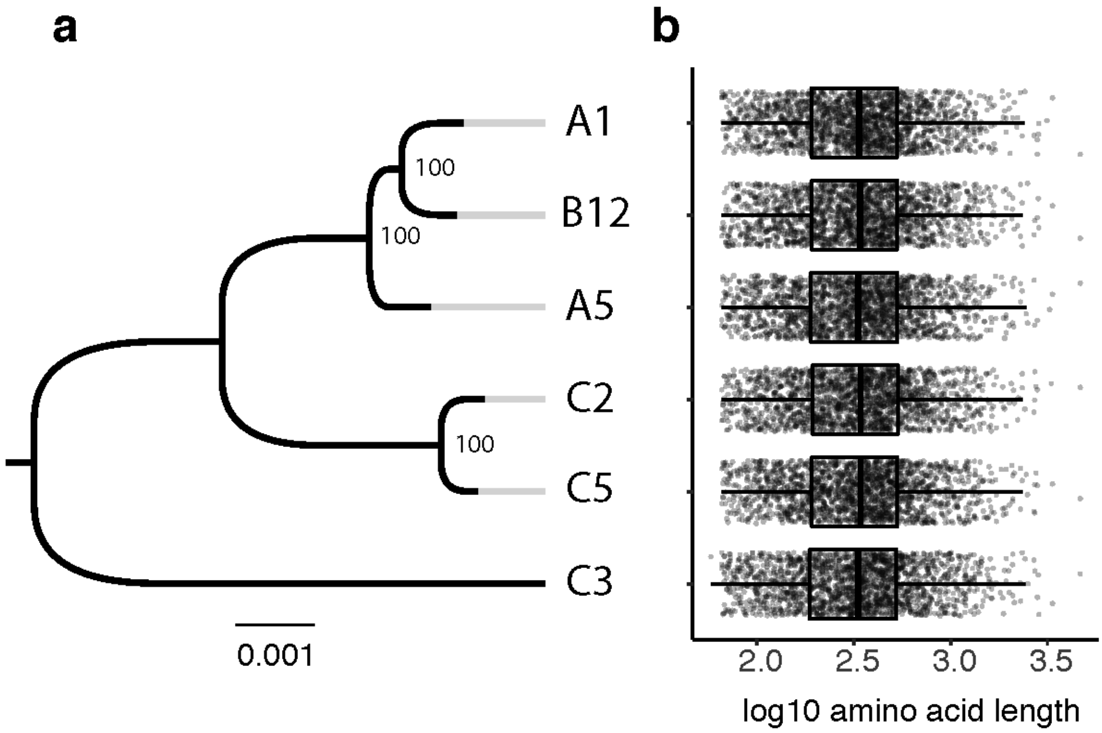

Supplementary Figure S1. Phylogeny and sequence lengths of conserved genes of six *Rhizophagus irregularis* isolates. (a) Phylogenetic distance of concatenated single copy UniProt accessions (n=1838) of *R. irregularis* isolates and computed by bootstrapping 1000 iterations with RAxML. The isolates A5, B12 and A1 clustered separately from C2 and C5, with roughly 0.0019 substitutions per site while proximity of C2 and C5 confirmed the very high degree of relatedness, whereas Isolate C3 was the most distant with > 0.005 substitutions per site **(b)** Box plots of individual amino acid lengths concatenated for alignment showing that the median sequence length (log10) did not vary significantly among the isolates, ranging from 2.521 in C3 to 2.535 in C2 and C5 (Supplementary Table 2).

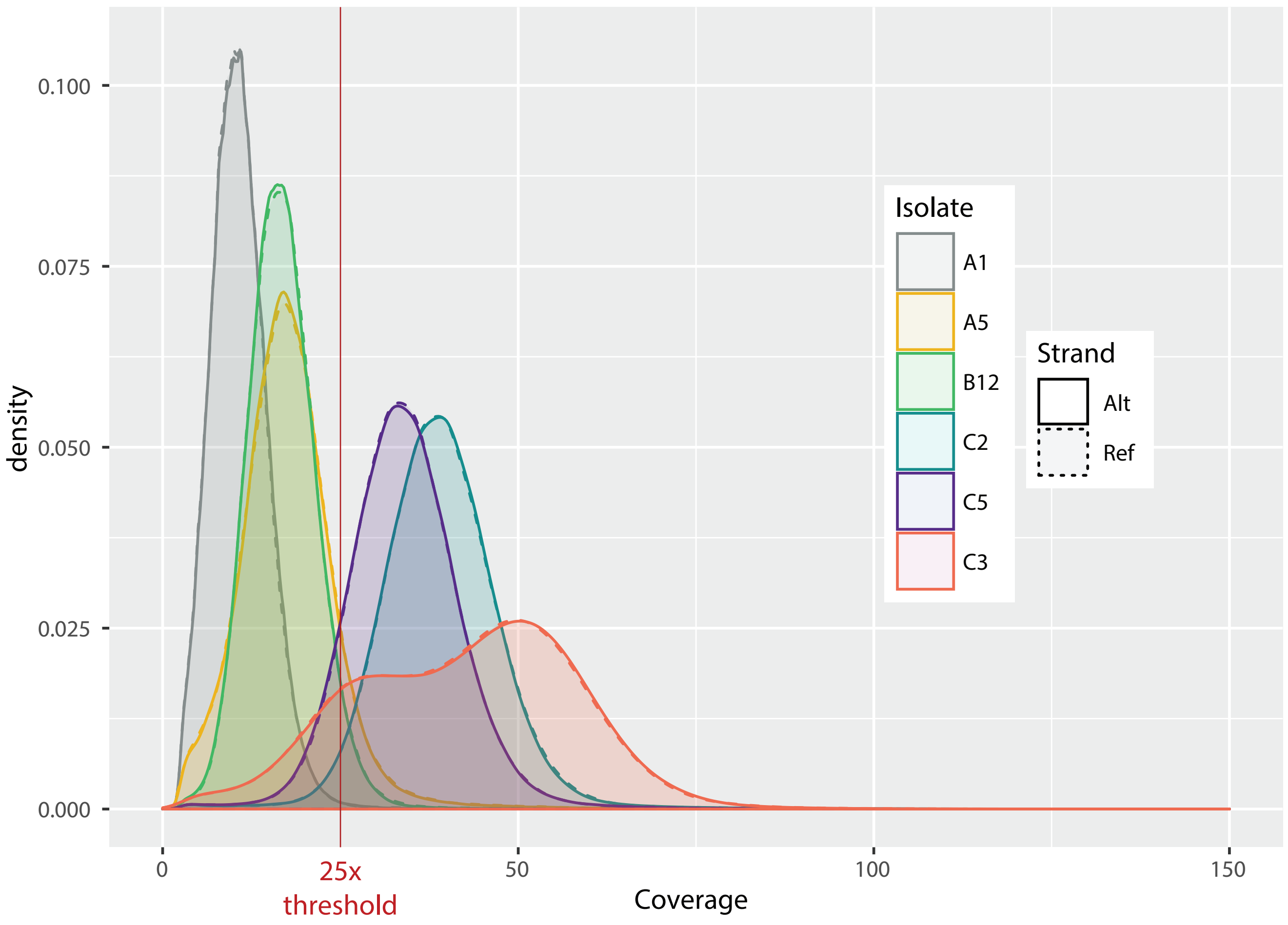

Supplementary Figure S2. Sequence read coverage distribution per DNA strand of six *R. irregularis* isolates. The 25x coverage/strand threshold is considered necessary for reliable 6mA calling.

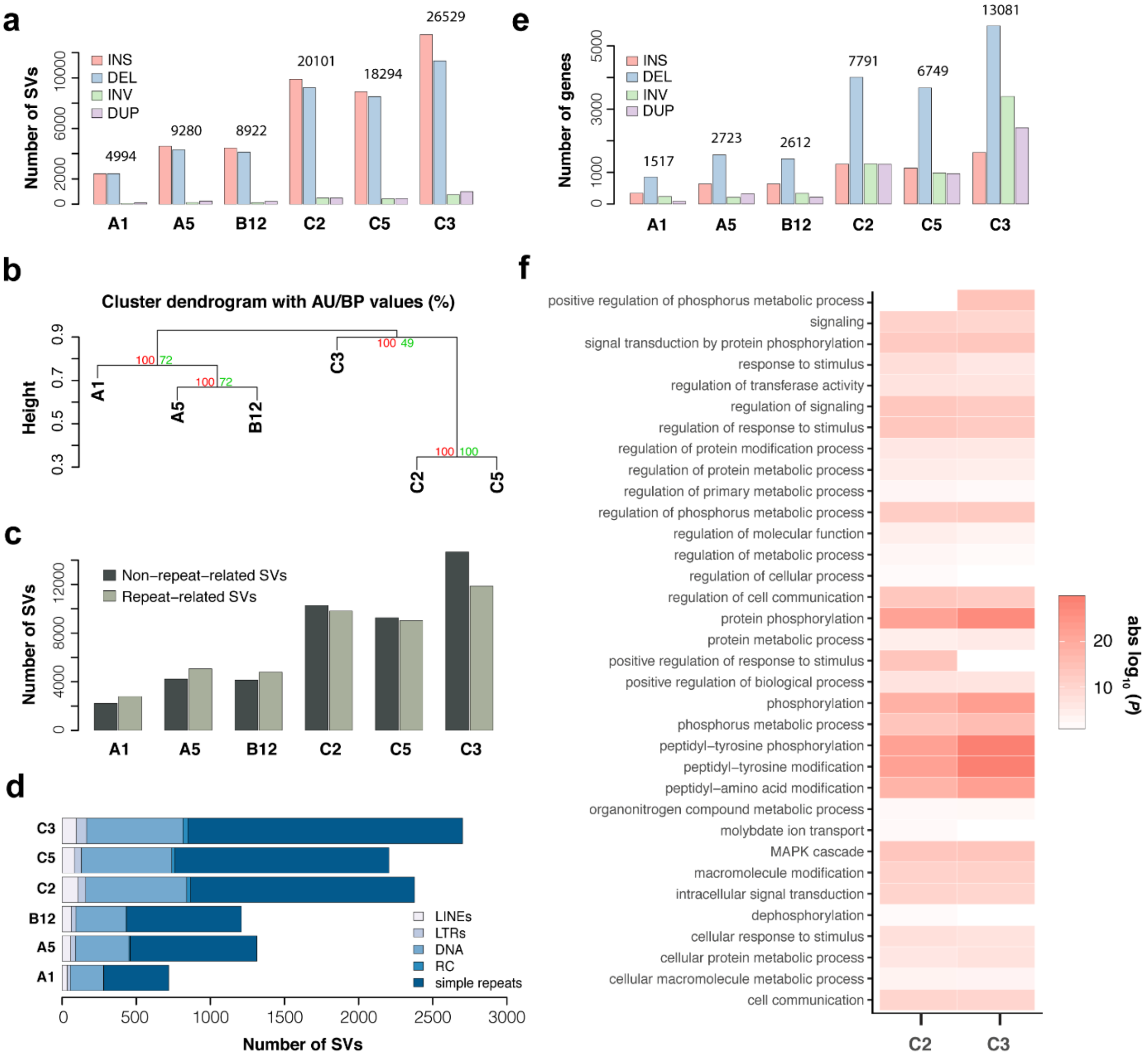

Supplementary Figure S3. Structural variation among genomes of six R. irregularis isolates compared to the R. irregularis DAOM197198 genome. **(a)** Number of SVs detected in the six genomes by SV type (INS: insertion; DEL: deletion; INV: inversion; DUP: duplication). **(b)** Hierarchical clustering of the six isolates based on the number of co-occurring SVs (AU: approximately unbiased probability shown in red; BP: bootstrap probability shown in green). **(c)** Number of repeat-related and non-repeat related SVs. **(d)** Number of SVs associated with known families of TEs (LINEs: long interspersed nuclear elements; LTRs: long terminal repeats retrotransposons; DNA: DNA transposons; RC: rolling circle transposons). **(e)** Number of genes where SV occurred. **(f)** Enriched GO categories assessed in the genes affected by SV in isolates C2 and C3. Define abs log 10 P.

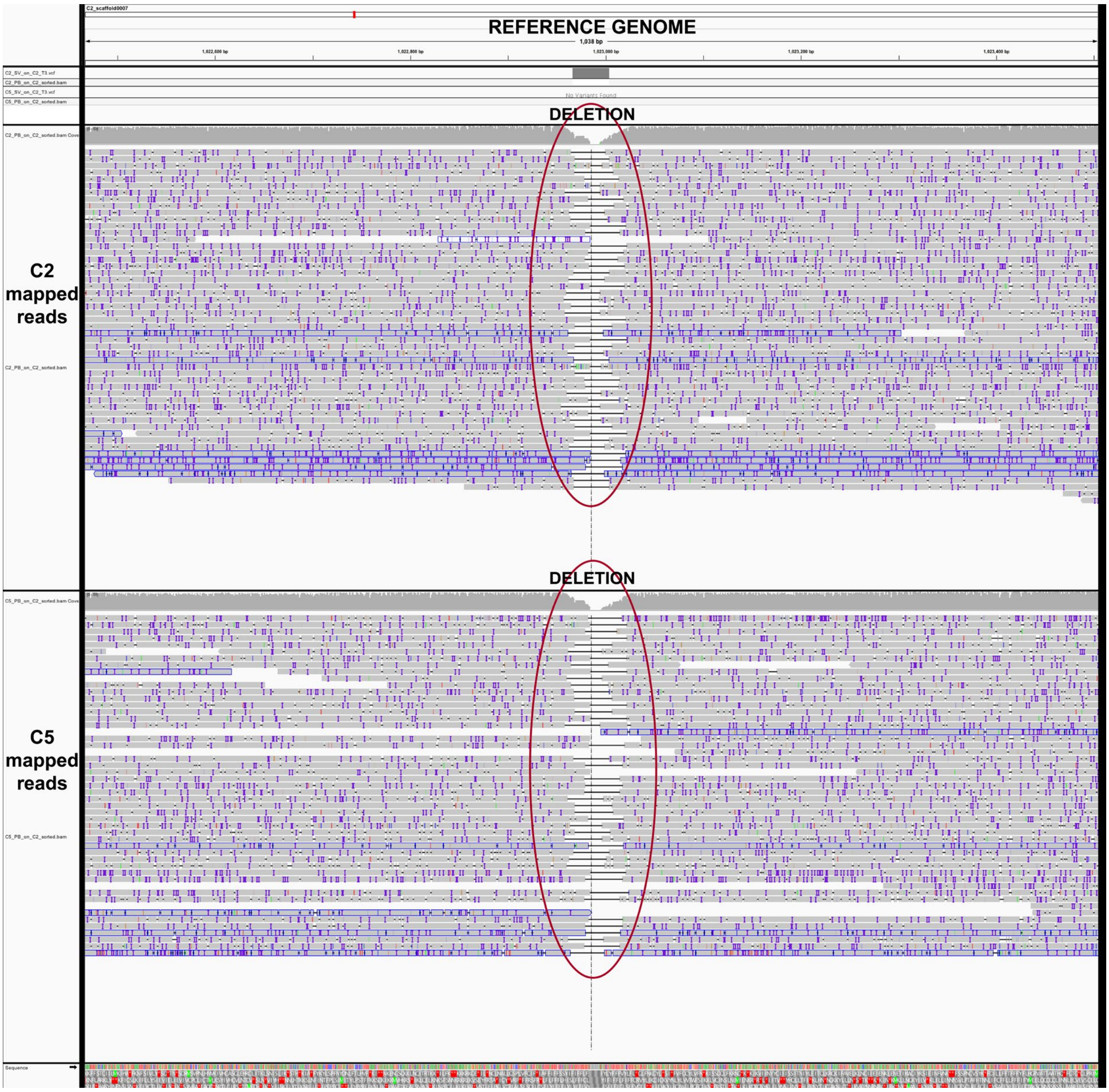

Supplementary figure S4. An example of the visualization of a discordant deletion between isolates C2 and C5 due to the lack of read support in C5. The deletion is clearly observed in the mapped reads of both isolates (a sudden drop of coverage in the highlighted genomic region). This SV was initially called in both isolates, but the read support value (RE) in isolate C5 was lower than a quality-filtering threshold (RE >15), while it was higher in isolate C2 (RE=18). Such sites were discarded after visual checks not considered as a structural variant between isolates C2 and C5.

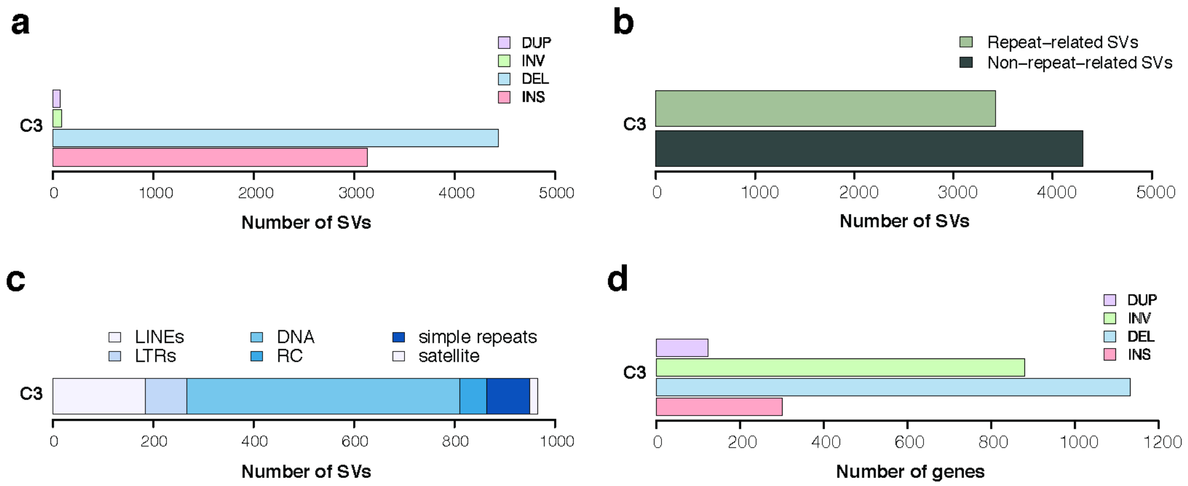

Supplementary Figure S5. Structural variation (SV) within the dikaryon R. irregularis isolate C3. (a) Number of SVs detected between the two nucleus genotypes. **(b)** Number of SVs related and non-related with the presence of repeated sequences. **(c)** Number of SVs associated with known families of TEs (LINEs: long interspersed nuclear elements; LTRs: long terminal repeats retrotransposons; DNA: DNA transposons; RC: rolling circle transposons). **(d)** Number of genes from the primary assembly that contained SVs in the haplotig.

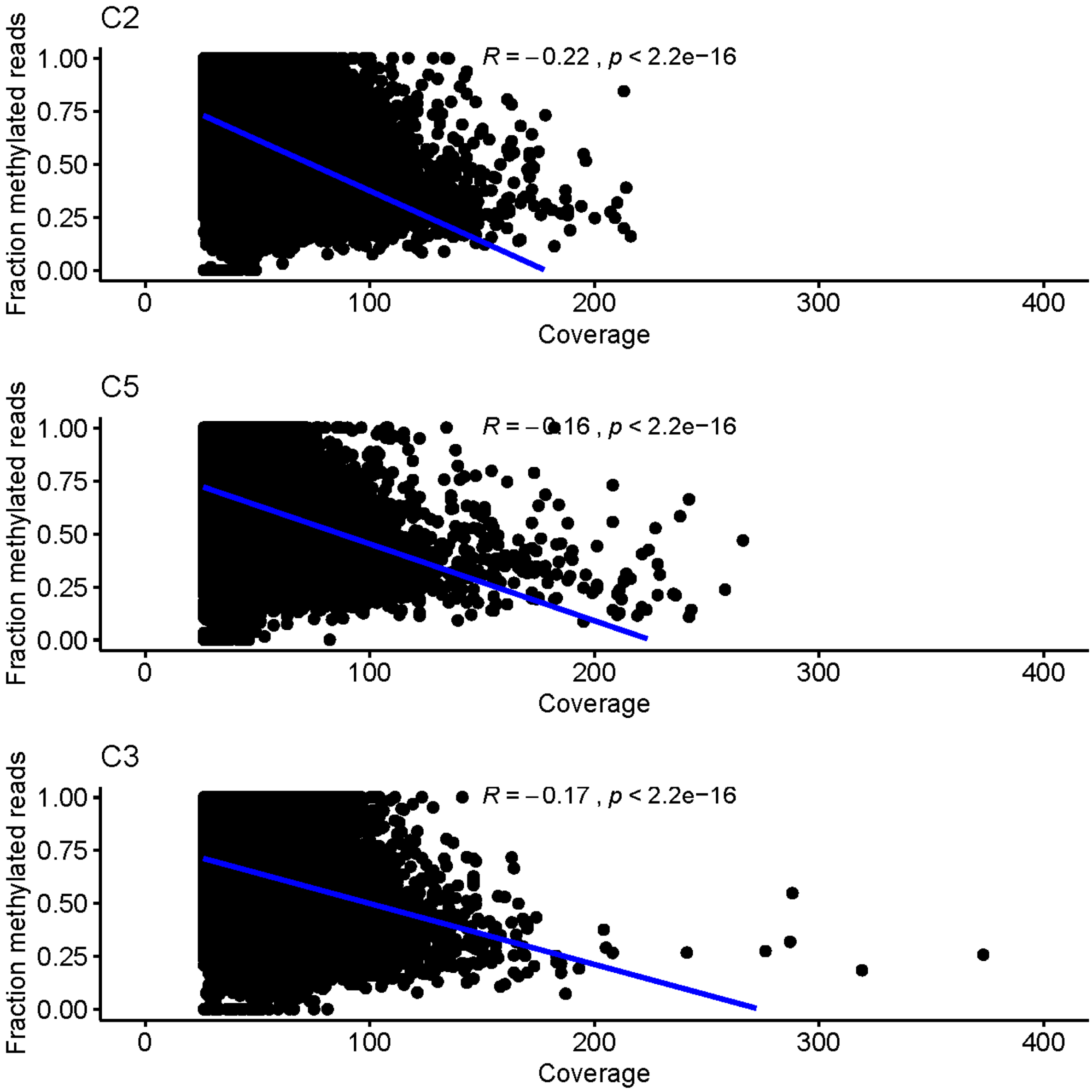

Supplementary figure S6. The relationship between the fraction of methylated to non-methylated reads and coverage in three *R. irregularis* isolates. The Pearson correlation coefficients showed a very weak negative linear relationship despite being significant in all three isolates, suggesting that coverage had a negligible effect on heterogeneity of 6mA methylation in the dataset.

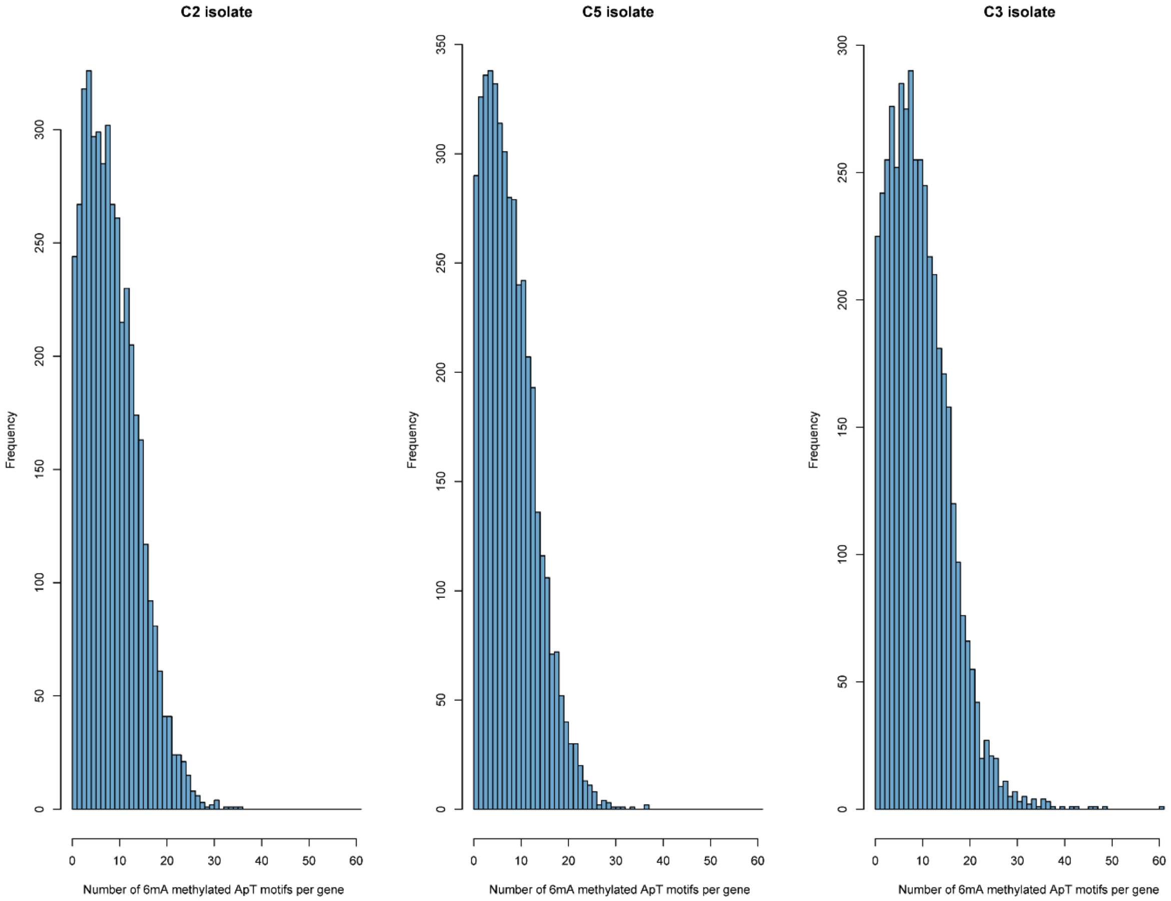

Supplementary figure S7. Distribution of number of 6mA methylated ApT motif per gene in 4398 commonly methylated genes in three *R. irregularis* isolates C2, C5 and C3.

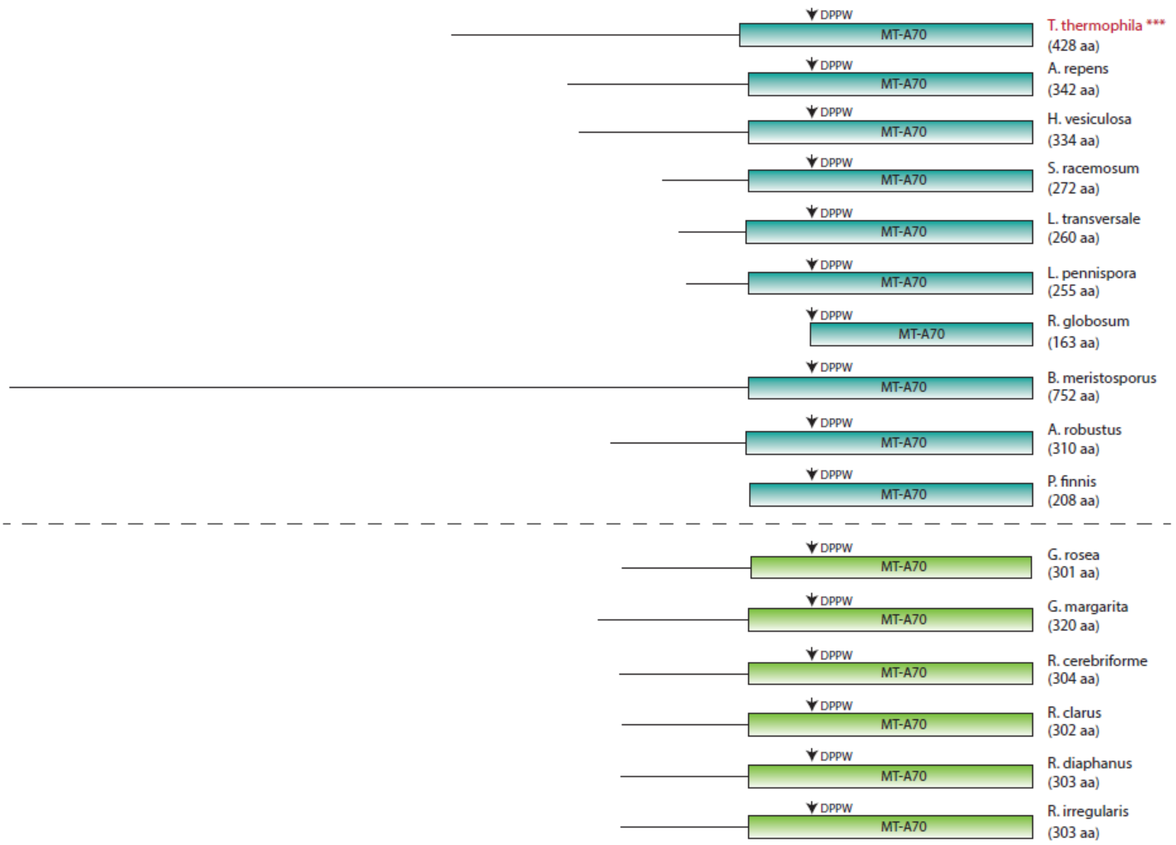

Supplementary figure S8. Conserved MT-A70 domain and catalytic motif (DPPW) in putative AMT1 MTases of EDF species. The arrow indicates the position of the DPPW motif within the sequence. The length of the protein sequences are indicated in brackets (number of aminoacids). *T. thermophila* AMT1 is shown as a reference (colored in red and marked with three asterisks). The dashed line separates the Glomeromycotina species (green) from other EDF species (blue).

Supplementary tables

Table S1.

Supplementary Table S1. Genome assembly and annotation statistics of six *R. irregularis* isolates Define what BUSCO completeness, fragmented and missing means.

	**A1**	**A5**	**B12**	**C2**	**C3**	**C5**
PacBio SMRT cells (RS II)	6	6	7	12	24	11
Number of scaffolds	151	392 (428 haplotigs)	121	123	232 (1084 haplotigs)	372
Assembled genome size (Mb)	147.98	115.47	146.58	159.8	159.63	166
Scaffold N50	25	61	25	20	30	36
GC%	28.09	27.39	28.09	28.5	27.94	27.64
N%	0.233	1.117	0.139	0.255	3.212	1.387
Coverage	20.52X	36X	30X	64X	79X	62X
Gene number	26293	28267	25711	27251	25670	27461
BUSCO completeness % **	92.1	86.9	95.9	95.9	95.1	95.5
BUSCO fragmented % **	4.1	3.8	2.4	2.1	3.1	2.1
BUSCO missing % **	3.8	9.3	1.7	2.0	1.8	2.4

Table S2.

Supplementary Table S2. One-way analysis of variance and Tukey multiple comparisons of means between isolates for log10(lengths) of proteins. 95% family-wise confidence level.

	Df	SumSq	MeanSq	Fvalue	Pr(>F)
**log10(lengths) of proteins**	5	0.1	0.0249	0.234	0.948
**residuals**	11022	1171.0	0.1062		

Comparison isolates (log10(lengths) of proteins)	Difference	Lower	Upper	Probability
A5-A1	-0.00302	-0.03367	0.027626	0.999766
B12-A1	0.000303	-0.03034	0.030949	1
C2-A1	0.002051	-0.02859	0.032697	0.999965
C3-A1	-0.00826	-0.03891	0.022384	0.972788
C5-A1	-0.00024	-0.03088	0.030409	1
B12-A5	0.003323	-0.02732	0.033969	0.999626
C2-A5	0.005071	-0.02557	0.035718	0.997108
C3-A5	-0.00524	-0.03589	0.025404	0.996616
C5-A5	0.002783	-0.02786	0.033429	0.999843
C2-B12	0.001749	-0.0289	0.032395	0.999984
C3-B12	-0.00856	-0.03921	0.022081	0.968181
C5-B12	-0.00054	-0.03119	0.030107	1
C3-C2	-0.01031	-0.04096	0.020332	0.930671
C5-C2	-0.00229	-0.03293	0.028358	0.99994
C5-C3	0.008025	-0.02262	0.038671	0.97605

Table S3.

Supplementary table S3. Number of structural variations (SV) detected in six *R. irregularis* isolates as compared to isolate DAOM 197198.

Number of structural variation events					
**Isolate**	**INSERTION**	**DELETION**	**INVERSION**	**DUPLICATION**	**TOTAL**
A1	2413	2413	57	111	4994
A5	4592	4308	126	254	9280
B12	4455	4119	123	225	8922
C2	9902	9226	485	488	20101
C5	8907	8518	425	444	18294
C3	13436	11347	750	996	26529

Table S4.

Supplementary table S4. The minimum, median and maximum lengths of each type of structural variant (SV) in the six *R. irregularis* isolates.

	Structural variant length											
	INSERTION			DELETION			INVERSION			DUPLICATION		
**Isolate**	**Min.**	**median**	**Max.**	**Min.**	**median**	**Max.**	**Min.**	**median**	**Max.**	**Min.**	**median**	**Max.**
A1	31	251	7930	31	352	177719	85	2078	837238	59	558	105948
A5	31	239	11244	31	410,5	304960	72	1108	237907	34	594,5	308333
B12	31	247	12094	31	363	104869	75	712	837238	51	555	231310
C2	31	239	22770	31	394	867212	66	1292	237908	35	868	871321
C5	31	249	13553	31	420	867212	71	1143	197265	35	841	871321
C3	31	196	14501	31	371	572715	69	2495,5	575919	37	2012	575869

Table S5.

Supplementary table S5. Number of structural variants (SV) related to the presence of transposable elements (TE) in the six *R. irregularis* isolates.

Number of transposable elements -enabled structural variants								
**Isolate**	**LINEs***	**LTRs***	**DNA***	**RC**	**Simple_repeat**	**Low_complexity**	**Unknown**	**Total**
A1	35	20	223	3	438	67	1445	2231
A5	58	32	361	8	855	124	2478	3916
B12	61	32	337	7	772	109	2437	3755
C2	108	51	681	26	1511	231	4812	7420
C5	83	47	609	22	1444	221	4397	6823
C3	96	70	649	34	1852	278	5361	8340

*LINEs: Long interspersed nucleotide elements; LTRs: Long terminal repeats; RC: Rolling Circle

Table S6.

Supplementary table S6. Number of genes directly containing structural variation (SV) in the six *R. irregularis* isolates.

Number of genes					
**Isolate**	**INSERTION**	**DELETION**	**INVERSION**	**DUPLICATION**	**TOTAL**
A1	342	856	237	82	1517
A5	633	1553	212	325	2723
B12	635	1426	336	215	2612
C2	1261	4007	1266	1257	7791
C5	1135	3673	983	958	6749
C3	1631	5638	3402	2410	13081

Supplementary table S7. Characterization of structural variation and 6mA ApT epigenetic marks occurring in genes involved in P transport, P metabolism, P responsive signalling, inositol polyphosphate biosynthesis and hydrolysis, PKA signalling, MAPK signalling, Tor signalling and sugar transport genes in *R. irregularis*. in isolates C2, C5 and C3. Isolates C2 and C5 are genetically indistinguishable. E: Exon; I: Intron and D: Downstream. Green represents the presence of structural variation and 6mA epigenetic marks and pink represents their absence.

Genes	Structural variation		6mA		
	C2	C3	C2	C5	C3
**Phosphate Transport**					
PT1					
PT2					
PT3	D	D			
PT4	E	E			
PT5					
PT6					
PHO87					
PHO90					
PHO91					
HA5					
Pho88					
**Phosphate Metabolism**					
Pho5					
ALP					
Vtc1					
Vtc2					
Vtc4	U	U			
Ppn1		D			
Gde1	D	D			
Vma10					
**Phosphate responsive signalling/Inositol polyphosphate biosynthesis/hydrolysis**					
VIP1	U	U			
PLC1					
ARG82					
KCS1	U				
IPK1					
DDP1		D			
Pho81					
Pho80	D	D			
Pho85					
**PKA signalling**					
TPK1					
Sch9	D	D			
NTH1		D			
TPS2					
TSL1	D	D			
Rim15					
Msn4					
Gis1	D				
SSA3		U			
HSP20					
SOD1	U	U			
SOD2					
BCY1					
**MAPK signalling**					
Mapk2					
Mek-2					
Mek-1					
NRC-1					
os-4		D			
os-5					
os-2					
Mik-1					
Mkc-1					
**Tor signalling**					
Tor2		I			
Gad8					
ste20					
Sin1					
Mip1					
**Sugar Transport**					
MST2	E, D	E, D			
MST3					
MST4					
SUC1					
E: Exon; I: Intron; U: Upstream; D: Downstream		Presence		Absence	

Supplementary notes References

1 Sedzielewska, K. A. *et al.* Estimation of the Glomus intraradices nuclear DNA content. *New Phytol* **192**, 794-797 (2011).

2 Tisserant, E. *et al.* Genome of an arbuscular mycorrhizal fungus provides insight into the oldest plant symbiosis. *Proc Natl Acad Sci U S A* **110**, 20117-20122 (2013).

3 Waterhouse, R. M. *et al.* BUSCO applications from quality assessments to gene prediction and phylogenomics. *Mol Biol Evol* (2017).

4 Savary, R. *et al.* A population genomics approach shows widespread geographical distribution of cryptic genomic forms of the symbiotic fungus Rhizophagus irregularis. *ISME J* **12**, 17-30 (2018).

5 Wyss, T. *et al.* Population genomics reveals that within-fungus polymorphism is common and maintained in populations of the mycorrhizal fungus Rhizophagus irregularis. *ISME J* **10**, 2514-2526 (2016).

6 Chen, E. C. H. *et al.* High intraspecific genome diversity in the model arbuscular mycorrhizal symbiont Rhizophagus irregularis. *New Phytol* **220**, 1161-1171 (2018).

7 Chen, E. C. *et al.* Single nucleus sequencing reveals evidence of inter-nucleus recombination in arbuscular mycorrhizal fungi. *Elife* **7** (2018).

8 Azevedo, C. & Saiardi, A. Eukaryotic Phosphate Homeostasis: The Inositol Pyrophosphate Perspective. *Trends Biochem Sci* **42**, 219-231 (2017).

9 Ezawa, T. & Saito, K. How do arbuscular mycorrhizal fungi handle phosphate? New insight into fine-tuning of phosphate metabolism. *New Phytol* **220**, 1116-1121 (2018).

10 Garcia, K. *et al.* Take a Trip Through the Plant and Fungal Transportome of Mycorrhiza. *Trends Plant Sci* **21**, 937-950 (2016).

11 Xie, X. *et al.* Arbuscular Mycorrhizal Symbiosis Requires a Phosphate Transceptor in the Gigaspora margarita Fungal Symbiont. *Mol Plant* **9**, 1583-1608 (2016).

12 Eskes, E., Deprez, M. A., Wilms, T. & Winderickx, J. pH homeostasis in yeast; the phosphate perspective. *Curr Genet* **64**, 155-161 (2018).

13 Helber, N. *et al.* A versatile monosaccharide transporter that operates in the arbuscular mycorrhizal fungus Glomus sp is crucial for the symbiotic relationship with plants. *Plant Cell* **23**, 3812-3823 (2011).

14 Wild, R. *et al.* Control of eukaryotic phosphate homeostasis by inositol polyphosphate sensor domains. *Science* **352**, 986-990 (2016).
