# Supplementary material for: The methylome of the model arbuscular mycorrhizal fungus, Rhizophagus irregularis, shares characteristics with early diverging fungi and Dikarya
Source: Commun Biol. 2021 Jul 22;4:901. doi: 10.1038/s42003-021-02414-5 (PMC8298701; doi:10.1038/s42003-021-02414-5)
Supplement: Supplementary file 7 — Description of Additional Supplementary Files [file 42003_2021_2414_MOESM7_ESM.pdf]

Description of Additional Supplementary Files

File name: Supplementary Data 1
Description: Source data for Figure 5c.

File name: Supplementary Data 2
Description: Source data for Figure 3a.

File name: Supplementary Data 3
Description: Source data for Figure 2a.

File name: Supplementary Data 4
Description: Source data for Figure 3c.
